# Effects of the PRIMROSE prevention trial of childhood obesity on parental self-efficacy

**DOI:** 10.1186/s12887-021-02862-2

**Published:** 2021-09-07

**Authors:** Nora Döring, Ata Ghaderi, Johanna Enö Persson, Per Tynelius, Finn Rasmussen, Benjamin Bohman

**Affiliations:** 1 Department of Global Public Health, Karolinska Institutet, Stockholm, Sweden; 2 Department of Clinical Neuroscience, Division of Psychology, Karolinska Institutet, Stockholm, Sweden; 3 Centre for Epidemiology and Community Medicine, Region Stockholm, Stockholm, Sweden; 4 Department of Clinical Neuroscience, Centre for Psychiatry Research, Karolinska Institutet, Stockholm, Sweden; 5 Stockholm Health Care Services, Region Stockholm, Stockholm, Sweden

## Abstract

**Background:**

Parental self-efficacy (PSE) has been suggested as a key factor for enabling parents to support children in the development of healthy dietary and physical activity behaviors and to prevent childhood obesity. However, studies of intervention effects on PSE are lacking. The present study involved a secondary analysis of data on PSE collected in a previous primary prevention trial of childhood obesity called the PRIMROSE trial. The trial involved a family-based intervention using motivational interviewing and principles of cognitive-behavioral therapy within a social-cognitive theory framework.

**Methods:**

In the PRIMROSE trial, parents and their children were randomly allocated to the intervention or usual care. In the present study, 928 mothers who responded to the Parental Self-Efficacy for Promoting Healthy Physical Activity and Dietary Behaviors in Children Scale (PSEPAD) at follow-up assessment were included. Data were analyzed using linear regression based on generalized estimating equations, with adjustment made for PSE at baseline.

**Results:**

At follow-up assessment, there was a statistically significant difference of 1.4 units, 95% CI [0.4, 2.4], *p* = 0.009, between the intervention and control conditions on the subscale of the PSEPAD concerning PSE for promoting healthy dietary behaviors in children. However, this difference was deemed as without clinical importance. On the total scale or other subscales of the PSEPAD there were no statistically significant differences in PSE between conditions.

**Conclusions:**

There was a statistically significant, but not clinically meaningful, intervention effect on PSE. However, because previous research repeatedly has shown positive associations of PSE with dietary and physical activity behaviors in children and that self-efficacy mediates behaviors, the construct may be important for influencing dietary and physical behaviors in children. Therefore, more research is warranted evaluating the effects of interventions on PSE in the context of childhood obesity prevention.

**Trial registration:**

Retrospectively registered 9 October 2013 at ISRCTN (ISRCTN16991919).

## Background

Childhood obesity is one of the greatest challenges of public health and early primary prevention has been suggested as the most promising strategy [1]. Dietary and physical activity (PA) behaviors are two main correlates of obesity that are established early in life and less malleable later on [2]. Parents play a vital role for the development of healthy dietary and PA behaviors [3]. Parents may not only act as “gate keepers” when it comes to making unhealthy food less available but are also the most important agents for establishing and maintaining healthy behaviors by being good role models and by providing an environment that facilitates desired behavior.

Parental self-efficacy (PSE) might be a key factor for enabling parents to play these vital roles successfully. The construct is defined as parents’ belief in their own capacity to influence their children’s behaviors. PSE is related to the general construct of self-efficacy, which is central to social-cognitive theory [4]. This theory argues that behavior is the result of a dynamic interplay between interpersonal, behavioral, and environmental factors. Self-efficacy is characterized as being specific to a domain of functioning, for example, work tasks or particular health behaviors, and has been found to mediate behaviors in different domains [5, 6]. Cross-sectional research has shown repeatedly that PSE is positively associated with children’s healthy dietary and PA behaviors (e.g., [7–10]). PSE may also play a central role in other parental domains. For example, it has been shown that the construct is associated with parental competence and psychological functioning, which in turn can be linked to depression, stress, role satisfaction, and coping [11]. In intervention research, applications of social-cognitive theory is often characterized by efforts to increase self-efficacy [5]. However, although social-cognitive theory is the most common theoretical framework in family-based childhood obesity prevention interventions [12], studies of intervention effects on PSE are lacking.

We have previously conducted a randomized controlled primary prevention trial of childhood obesity called the PRIMROSE trial [13]. The intervention was family-based and used motivational interviewing (MI) and principles of cognitive-behavioral therapy employed within a social-cognitive framework to influence parents to promote healthy dietary and PA behaviors in their children. However, the results of the trial showed no intervention effects on weight-related measures (i.e., body mass index [BMI], waist circumference, and prevalence of overweight) in children or mothers or on children’s or mothers’ PA behaviors, but small effects on dietary behaviors in children and mothers. To explore the lack of effects of the previous trial and inform future trials of childhood obesity prevention, the present study involved a secondary analysis of data of the PRIMROSE trial to investigate the effects of the intervention on PSE.

## Methods

### The PRIMROSE trial

The protocol of the PRIMROSE trial, including detailed information about the design, methods, and intervention components, has been published elsewhere [14]. In brief, the PRIMROSE trial was a population-based cluster-randomized controlled primary prevention trial of childhood obesity that started in 2008 and was completed in 2015. The trial was conducted within routine child health services in Sweden and included 1369 children and their parents. Nurses at child health centers allocated to the intervention condition used MI and principles of cognitive-behavioral therapy to promote healthy dietary and PA behaviors. The intervention comprised of nine sessions starting when the children were 9 months of age and continued for their first 4 years. During the same period, participants in the control condition received usual care (i.e., regular health check-ups).

### Participants

In the present study, 928 mothers who responded to a PSE measure at follow-up assessment when children were 4 years of age were included (*n* = 388 in the intervention condition and *n* = 540 in the control condition). For characteristics of participants at baseline in the PRIMROSE trial, see Table 1.

**Table 1:** Baseline Characteristics of Participating Parents

	(*n* = 388)	(*n* = 540)	*p*
	*n* (%) / Mean (*SD*)	*n* (%) / Mean (*SD*)	
Gender, female	388 (100)	540 (100)	
Age (years)	30.3 (5.1)	29.6 (4.9)	0.032
Education			0.003
Primary	7 (1.8)	18 (3.3)	
Secondary	115 (29.6)	203 (37.5)	
Post-secondary	266 (68.6)	319 (59.6)	
Born in Sweden	373 (96.1)	495 (91.7)	0.006
BMI (kg/m^2^)	24.5 (4.5)	24.8 (4.9)	0.424
Waist circumference (cm)	83.7 (11.4)	84.2 (11.7)	0.590
Perceived general health			0.566
Very good	93 (23.9)	129 (23.4)	
Good	215 (55.4)	314 (58.2)	
Fairly good	69 (17.8)	84 (15.6)	
Bad or very bad	11 (2.4)	13 (2.8)	

*Note*. *BMI* Body mass index

### Assessment

PSE was assessed using the Parental Self-Efficacy for Promoting Healthy Physical Activity and Dietary Behaviors in Children Scale (PSEPAD; 15), a 14-item measure with three subscales of relevance to early childhood obesity prevention: PSE for promoting healthy dietary behaviors in children (Subscale 1), PSE for limit-setting of unhealthy dietary and PA behaviors in children (Subscale 2), and PSE for promoting healthy PA behaviors in children (Subscale 3). Participants assessed the strength of their efficacy beliefs on an 11-point response scale ranging from 0 (*Not at all*) to 10 (*To a very high degree*). Exploratory and confirmatory factor analyses have yielded a three-factor structure of the PSEPAD, corresponding to the subscales, providing support of construct validity [15]. Further, the PSEPAD has shown high internal consistency, with Cronbach’s *α* = 0.87 for the total scale, *α* = 0.75 for Subscale 1, *α* = 0.76 for Subscale 2, and *α* = 0.80 for Subscale 3, a high two-week test-retest reliability of Pearson’s *r* = 0.82, as well as adequate discriminant validity [15]. In the present study, *α* = 0.89 for the total scale, *α =* 0.80 for Subscale 1, *α* = 0.79 for Subscale 2, and *α* = 0.87 for Subscale 3.

### Data analysis

Linear regression based on generalized estimating equations with robust variance estimates, taking into account the cluster-randomized trial design, was used to investigate intervention effects on the PSEPAD total scale and its subscales. Adjustment for PSE at baseline was made in the analyses. Because there were statistically significant baseline differences between intervention and control conditions on parental age, education, and whether they were born in Sweden or not (see Table 1), adjustments for these variables were also made in the analyses. However, results adjusted for age, education, and Sweden as country of birth were similar to unadjusted results; thus, results are presented without these adjustments. In addition, stratified analyses were conducted by including an interaction term between the intervention condition and parental obesity (BMI ≥ 30 vs BMI < 30), age (≥ 35 years vs < 35 years), education (post-secondary vs other), and country of birth (Sweden vs other). However, these analyses were not statistically significant (*p*s = 0.098–0.999) and are not presented.

## Results

At follow-up assessment, there was a statistically significant difference of 1.4 units, 95% CI [0.4, 2.4], *p* = 0.009 between intervention and control conditions on the subscale of the PSEPAD concerning PSE for promoting healthy dietary behaviors in children. However, this small difference was deemed as without clinical importance. On the total scale or other subscales, there were no statistically significant differences in PSE between conditions. See Table 2 for estimated means, standard errors, and results of regression analyses comparing the intervention and control conditions on the PSEPAD.

**Table 2:** Estimated Means, Standard Errors, and Results of Regression Analyses on the PSEPAD at Follow-Up Assessment

	Intervention (*n* = 388)	Control (*n* = 540)			
	Est. Mean (*SE*)	Est. Mean (*SE*)	Mean Δ [95% CI]	p*	p**
Total scale	123.9 (0.99)	122.2 (0.78)	1.7 [−0.8, 4.1]	0.184	0.142
Subscale 1	52.4 (0.40)	51.0 (0.32)	1.4 [0.4, 2.4]	0.007	0.009
Subscale 2	47.2 (0.47)	47.0 (0.34)	0.2 [−1.0, 1.3]	0.780	0.786
Subscale 3	24.4 (0.20)	24.2 (0.23)	0.2 [−0.4, 0.8]	0.562	0.849

*Note*. *PSEPAD* Parental Self-Efficacy for Promoting Healthy Physical Activity and Dietary Behaviors in Children Scale, Subscale 1 = Parental self-efficacy for promoting healthy dietary behaviors in children, Subscale 2 = Parental self-efficacy for limit-setting of unhealthy dietary and physical activity behaviors in children, Subscale 3 = Parental self-efficacy for promoting healthy physical activity behaviors in children

*Unadjusted, **Adjusted for parental self-efficacy at baseline

## Discussion

The present study involved a secondary analysis of data of the PRIMROSE trial, a previous primary prevention trial of childhood obesity [13]. Specifically, the effects of the intervention on PSE for promoting healthy dietary and PA behaviors in children at follow-up assessment were investigated. Results showed a statistically significant difference of 1.4 units between the intervention and control conditions on the subscale of the PSEPAD concerning PSE for promoting healthy dietary behaviors in children. There were no statistically significant differences on the total scale or other subscales.

To some extent, these findings mirror the results of the PRIMROSE trial, which found small statistically significant effects on dietary behaviors in children and mothers, but no effects on children’s or mothers’ PA behaviors [13]. A possible explanation of the differential effects of dietary and PA behaviors in the PRIMROSE trial is that the intervention focused more on dietary behaviors. Another explanation might be that whereas PA behaviors in children were objectively assessed using accelerometry, dietary behaviors were reported by mothers; thus, responses may have been subjected to recall bias or social desirability bias [13]. The results of the present study are similar to a study by Nyberg and colleagues [16], which also compared PSE between intervention and control conditions in a childhood obesity prevention trial conducted in a school setting in Sweden and assessed PSE using a similar measure. However, in this study, there were not any differences on PSE between conditions, neither on the total scale nor subscales, and neither at follow-up assessment nor 6 months following intervention completion.

The difference of 1.4 units between the intervention and control conditions concerning PSE for promoting healthy dietary behaviors in children was statistically significant; however, although it has not been empirically evaluated, such a small difference on a measure with scores ranging from 0 to 140 is hardly of any clinical relevance. Possible explanations of the lack of a clinically meaningful intervention effect on PSE may be similar to the explanations of the lack of intervention effects on weigh-related measures and PA behaviors in the PRIMROSE trial [13]. First, there are some indications that the intervention may not have been fully implemented as intended. Evaluations of nurses’ proficiency in MI using the Motivational Interviewing Treatment Integrity Code [17] were conducted following training and supervision in MI and at three occasions during the trial when nurses had received additional supervision. Results showed that nurses did not acquire skillfulness in MI, which was the main intervention component, despite extensive training and supervision [18, 19]. This may have resulted in a failure also to increase PSE in parents.

A second possible explanation is that the intervention dose or intensity of the intervention (i.e., nine sessions over 4 years) may not have been sufficient to produce change on most outcomes, thus also on PSE. Finally, the PSEPAD may not have been sufficiently sensitive to change, despite being developed according to recommendations [20]. Sensitivity to change of PSE measures is of particular relevance to prevention interventions, especially primary prevention interventions, because they may involve participants with high levels of PSE already at baseline. If a measure has low sensitivity, increases in PSE from an already high level will not show on the measure. However, the sensitivity of the PSEPAD was not assessed.

Strengths of the present study include the large sample size and the use of a validated measure of PSE (i.e., the PSEPAD). However, the PSEPAD has not been validated for parents with infants; thus, PSE at baseline should be interpreted with caution. Other limitations include the lack of MI fidelity and issues of intervention dose and intensity.

## Conclusions

The present study showed that there was a statistically significant, but not clinically meaningful, intervention effect on PSE for promoting healthy dietary behaviors in children in a previously conducted primary prevention trial of childhood obesity. Previous research has repeatedly shown positive associations of PSE with dietary and PA behaviors in children and that self-efficacy mediates behaviors in different domains of functioning. Thus, the construct may be important for influencing dietary and PA behaviors in children. However, there are few studies investigating intervention effects on PSE, despite social-cognitive theory being the most common theoretical framework in childhood obesity prevention trials. Therefore, more research is warranted evaluating the effects of interventions on PSE, and PSE as a potential mediator of intervention effects, in the context of childhood obesity prevention.

## Data Availability

The dataset analyzed during the current study is not publicly available due to issues of confidentiality but are available from the corresponding author on reasonable request.

## Abbreviations

BMI: Body mass index
MI: Motivational interviewing
PA: Physical activity
PSE: Parental self-efficacy
PSEPAD: Parental Self-Efficacy for Promoting Healthy Physical Activity and Dietary Behaviors in Children Scale
